# Intra-cavitary radiotherapy for surgically resected brain metastases: a comprehensive analysis including an individual patient data meta-analysis of intraoperative radiotherapy (IORT) and brachytherapy (IBT)

**DOI:** 10.1007/s11060-025-05227-2

**Published:** 2025-09-17

**Authors:** Alexandru Guranda, Erdem Güresir, Arne Mathias Ruder, Frank Anton Giordano, Johannes Wach

**Affiliations:** 1 Department of Neurosurgery, University of Leipzig Medical Center, Leipzig University, Liebigstraße 20, 04103 Leipzig, Germany; 2 Comprehensive Cancer Center Central Germany, Partner Site Leipzig, 04103 Leipzig, Germany; 3 Department of Radiation Oncology, Medical Faculty Mannheim, University Medicine Mannheim, Heidelberg University, 68167 Mannheim, Germany; 4 DKFZ Hector Cancer Institute at the University Medical Center Mannheim, 68167 Mannheim, Germany; 5 Mannheim Institute for Intelligent Systems in Medicine (MIISM), Medical Faculty, Heidelberg University, 68167 Mannheim, Germany

**Keywords:** Brain metastases, Intraoperative radiotherapy, Brachytherapy, Local control, Radiation necrosis, Individual patient data meta-analysis

## Abstract

**Background:**

Surgical resection followed by adjuvant radiotherapy is a standard approach for brain metastases (BM). Intracavitary radiotherapy techniques—namely intraoperative radiotherapy (IORT) and brachytherapy (IBT)—have gained attention as alternatives to stereotactic radiotherapy, potentially reducing neurotoxicity and treatment delays. However, robust comparative data remain scarce.

**Methods:**

We performed a systematic meta-analysis including both conventional and reconstructed individual patient data (IPD) from studies reporting outcomes after intracavitary radiotherapy post-BM resection. Primary endpoint was local control rate (LCR); secondary endpoints included overall survival (OS), distant brain control (DBC), radiation necrosis (RN), and leptomeningeal disease (LMD). IPD was reconstructed from published Kaplan-Meier curves. Survival and incidence outcomes were pooled using random-effects models in R.

**Results:**

Twenty-three studies with 858 patients were analyzed. The 1-year LCR was 96% (95% CI: 94–98%) for IORT and 95% (95% CI: 92–97%) for IBT. Median OS in patients who underwent IORT was 39.1 months (95% CI: 22.0–59.5), and 15.9 months (95% CI: 12.6–19.9) in whose who underwent IBT, respectively (*p* = 0.004; HR 0.64). IORT was associated with lower RN (4% vs. 7%) and LMD (6% vs. 9%). The 1-year DBC rate was higher for IORT (57%) than IBT (48%).

**Conclusions:**

Intracavitary radiotherapy yields excellent local control after BM resection. This IPD meta-analysis provides the most comprehensive evidence to date and supports further prospective evaluation of IORT in neuro-oncological care.

**Graphical abstract:**

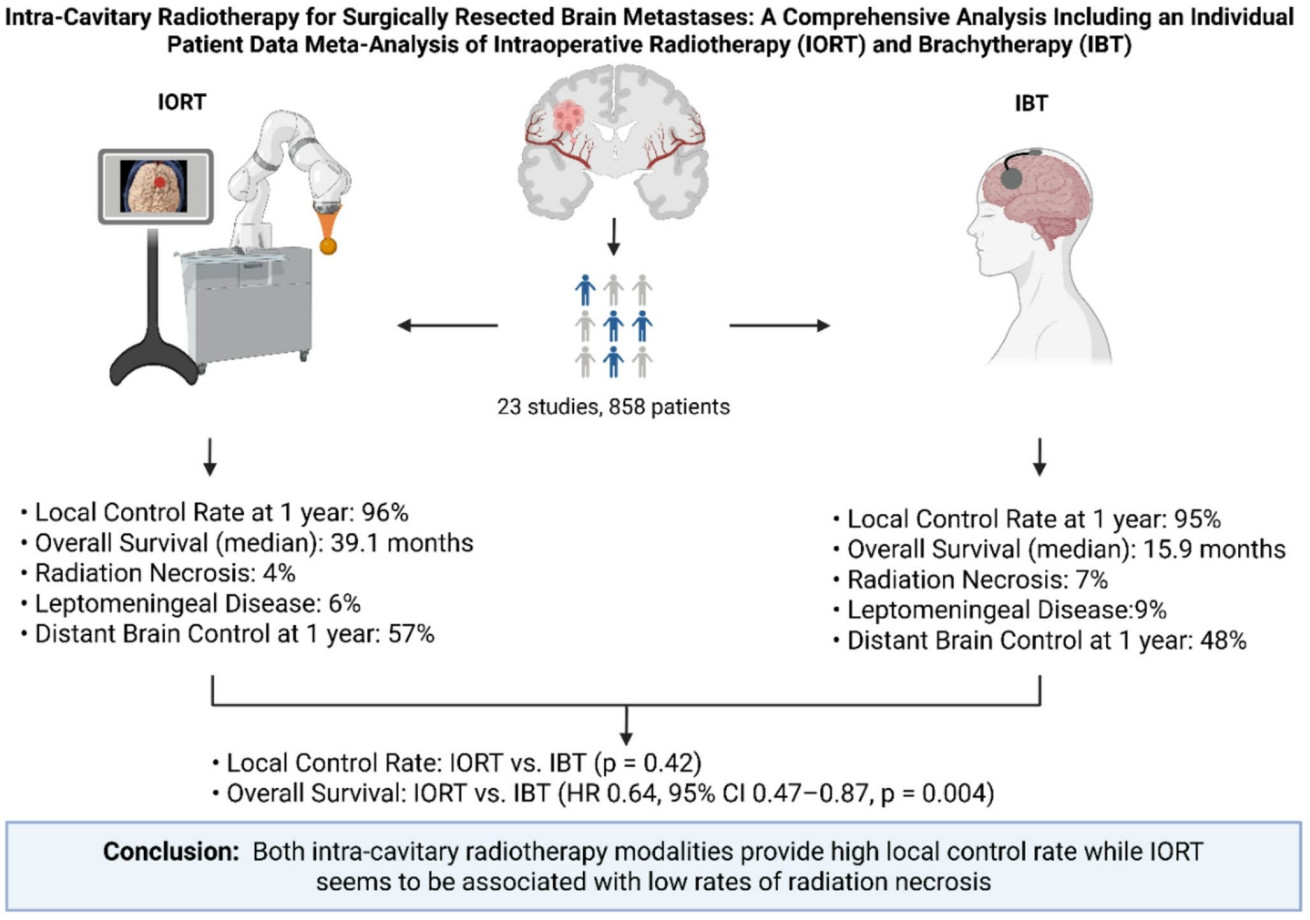

**Supplementary Information:**

The online version contains supplementary material available at 10.1007/s11060-025-05227-2.

## Introduction

Brain metastases (BM) are the most common intracranial malignancy in adults, affecting ~ 2% of all cancer patients and up to 12.1% of those with metastatic disease at diagnosis [1, 2]. Despite advances in systemic therapy, local treatment remains essential. For patients with few accessible BM, maximal safe resection is standard, particularly with symptomatic lesions, mass effect, or radioresistant tumors. Surgery allows cytoreduction and definitive histology [3, 4]. However, residual microscopic disease often leads to high recurrence rates (46–60%) without adjuvant therapy [5, 6]. Thus, postoperative radiotherapy is commonly used to improve local control [7].

Traditionally, whole-brain radiotherapy (WBRT) was employed, but despite improved local control, it offers limited survival benefit (median 3–6 months) [8] and poses risks of neurocognitive decline [9, 10]. This has led to a shift toward local therapies like stereotactic radiosurgery (SRS), intraoperative radiotherapy (IORT), and intracavitary brachytherapy (IBT), which better preserve cognitive function [7].

IORT and IBT deliver localized radiation during or immediately after resection [11, 12]. IORT uses low-energy X-rays or electron beams; IBT implants radioactive sources into the cavity. Both reduce treatment delays, minimize exposure to healthy brain tissue, and show promising 1-year local control rates of 85–96% [11].

Given their increasing clinical use, this meta-analysis evaluates the efficacy and safety of IORT and IBT following BM resection. Beyond aggregate data synthesis, we reconstruct individual patient data (IPD) to refine survival estimates and directly compare IORT and IBT. This study aims to provide a comprehensive assessment of their impact on local control, distant brain failure, overall survival, and treatment-related toxicity—specifically radiation necrosis and leptomeningeal disease.

## Methods

### Search strategy and data collection

The present study was prospectively registered in the international Prospective Register of Systemic Reviews (ID: CRD420251026983). We systematically searched PubMed and Google Scholar (1999–Jan 2025) for studies on IORT or IBT in resected brain metastases. Keywords included “brain metastasis” AND “IORT,” “brachytherapy,” or “intracavitary radiation” [MeSH]. Study design and language were initially unrestricted, but non-English papers were later excluded. References of relevant articles were hand-searched. After duplicate removal, titles and abstracts were screened; full texts were reviewed based on predefined criteria. PRISMA guidelines were followed (see supplementary material 1). Studies were excluded if they lacked clinical outcomes, involved pediatric patients (< 18 years), or included < 5 cases. Both retrospective and prospective studies were eligible. A PRISMA flowchart documented identification, eligibility, and inclusion, with reasons for exclusion (e.g., missing outcome data or overlapping cohorts). Supplementary material 2 shows the study protocol.

## Inclusion criteria

Eligible studies met the following criteria.

(1) Population: Patients with histologically confirmed BM treated surgically. Studies on primary brain tumors or non-surgical cases were excluded. Both newly diagnosed and recurrent cases (post-SRS/WBRT) were eligible if cavity-directed radiotherapy was used. (2) Intervention: Localized radiotherapy via IORT (intraoperative 50-kV X-ray or electrons) or IBT (e.g., I-125 seeds, HDR catheters, balloon-based devices). (3) Outcomes: Required endpoints were LCR, OS, and DBC. LMD and RN were noted when reported. (4) Study design: Retrospective and prospective studies were eligible. Abstract-only studies were accepted if sufficient data were available; case reports with < 5 patients were excluded.

Patients receiving cavity irradiation either after prior radiotherapy (salvage) or in a de novo adjuvant setting were included.

## Quality assessment

Study quality and bias risk were assessed using the NIH Quality Assessment Tool for observational cohort and cross-sectional studies [13]. Studies were rated independently by two reviewers as “Good,” “Fair,” or “Poor” based on clarity, population definition, participation, outcome measurement, follow-up, and bias. Discrepancies were resolved by consensus.

Prospective studies with clear outcomes scored higher; retrospective studies with unclear methodology scored lower. Limitations included non-randomization, selection bias, and heterogeneity. Overlapping cohorts were resolved by prioritizing the most comprehensive datasets. Grey literature and abstracts were screened to reduce publication bias and account for underreported negative results.

## Data extraction and endpoints

Data were extracted via a standardized form, covering study design, patient demographics (sample size, age, KPS), tumor characteristics, treatment modality (IORT vs. IBT), and follow-up. Primary outcomes included LCR, OS, DBC, LMD, and RN. LCR was defined as absence of cavity recurrence, typically at 1 year; if not reported, it was derived from failure rates. OS was defined from surgery to death. DBC was calculated as 1 minus distant brain failure.

LMD and RN were included if reported; RN was recorded as 0% if explicitly absent. Wound complications were also noted.Several studies provided Kaplan-Meier (KM) curves for LCR and OS. Individual patient data were reconstructed from KM plots using Guyot’s method and DigitizeIt [14]. Curves and risk tables were generated via the Liu et al. approach [15], and reconstructed survival metrics were cross-checked with published data for accuracy.

### Statistical analysis

We used the R package IPDfromKM in R Studio to digitize and reconstruct time-to-event data, stratifying KM curves by treatment (IORT vs. IBT) [15]. Survival analyses were performed with survival and survminer (log-rank test, Cox regression for HRs with 95% CIs).

One-year LCR, DBC, LMD, and RN rates were pooled using a DerSimonian–Laird random-effects model and visualized with forest plots. Heterogeneity was assessed via I² and Q-test; significance was set at *p* < 0.05.

## Results

### Study selection and characteristics

**Fig. 1:**
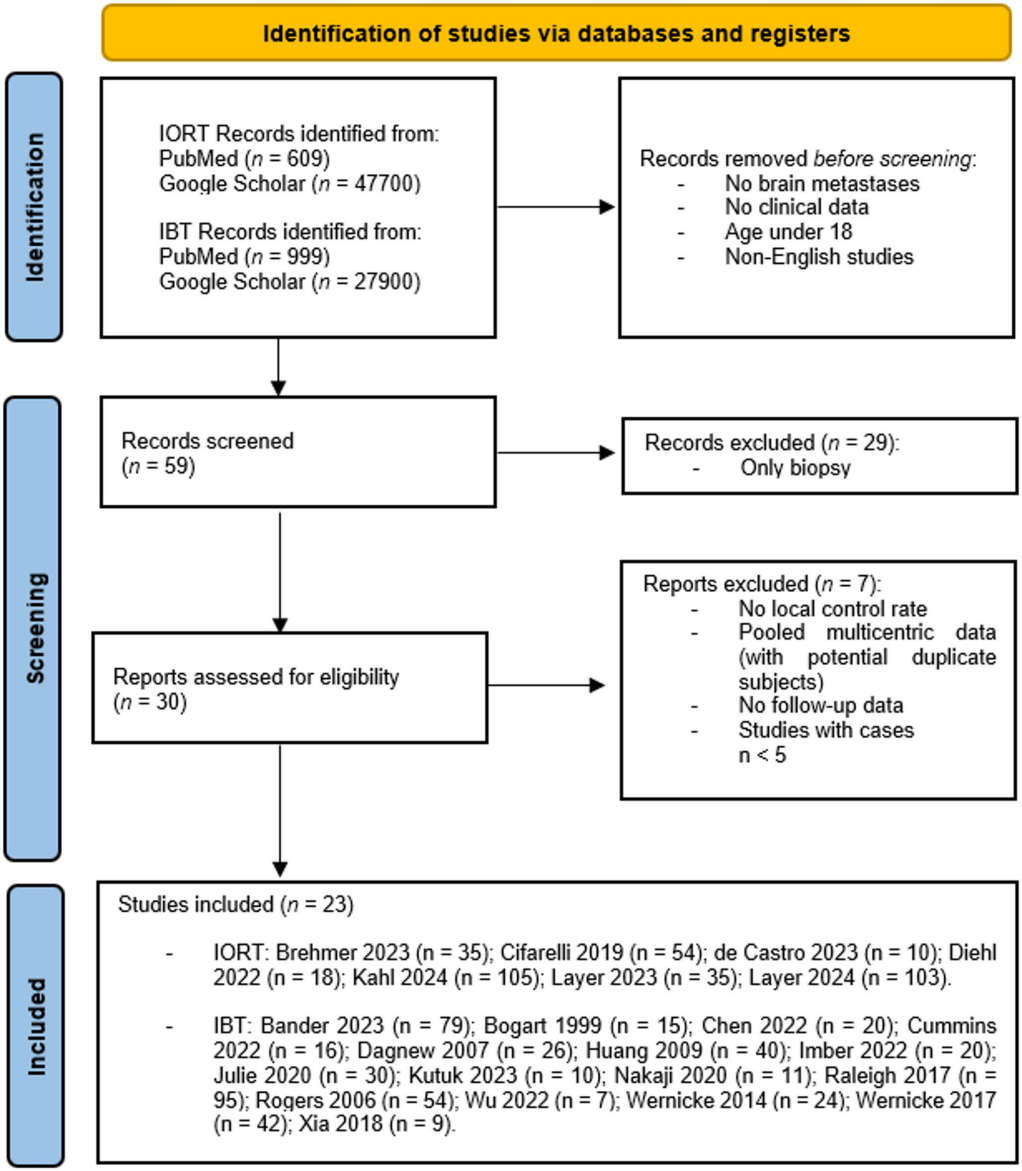
PRISMA flowchart for study selection

The systematic review yielded 77,208 records (47,700 IORT and 27,900 IBT entries via Google Scholar; 609 IORT and 999 brachytherapy entries via PubMed; Fig. 1). After screening, 59 articles remained, with 23 studies meeting all inclusion criteria. The final cohort included 16 retrospective and 7 prospective single-arm studies (1999–2024), covering 858 patients treated with IORT or IBT after BM resection. Supplementary Table 1 summarizes study design, patient characteristics, treatment, and follow-up.

Sixteen studies investigated IBT [16–31], and seven focused on IORT [32–38]. Across 23 studies, the median patient age was 60 ± 3.46 years; Bogart et al. did not report age [17]. Karnofsky Performance Status (KPS) was inconsistently reported; 13 studies documented a median KPS of 80 ± 6.50 [17–21, 23–27, 35, 37, 38]. Follow-up data were available in 19 studies, with a pooled median of 12.6 ± 13.95 months.

Lung cancer (NSCLC = 224, SCLC = 4, unspecified = 176; total *n* = 404) was the most frequent histology, followed by melanoma (*n* = 123), breast (*n* = 96), renal (*n* = 44), and gastrointestinal cancers (*n* = 41). Key characteristics of the included studies are summarized in supplementary Table 1.

## Overall survival

OS was evaluated as a secondary endpoint of this meta-analysis. The survival rate at one year was 58%, based on data from studies that reported this outcome [16, 18, 20–23, 27–29, 33–38]. OS data from individual studies were as follows: Brehmer et al. (37.4 months) [32], Wu et al. (11.07 months) [30], Nakaji et al. (9.3 months) [25], Xia et al. (10.3 months) [31], Bogart et al. (14 months) [17], Raleigh et al. (12 months) [26], Dagnew et al. (17.8 months) [20], Cummins et al. (17.9 months) [19], and Julie et al. (66.7%) [23] Kutuk et al. [24] did not report OS.

**Fig. 2:**
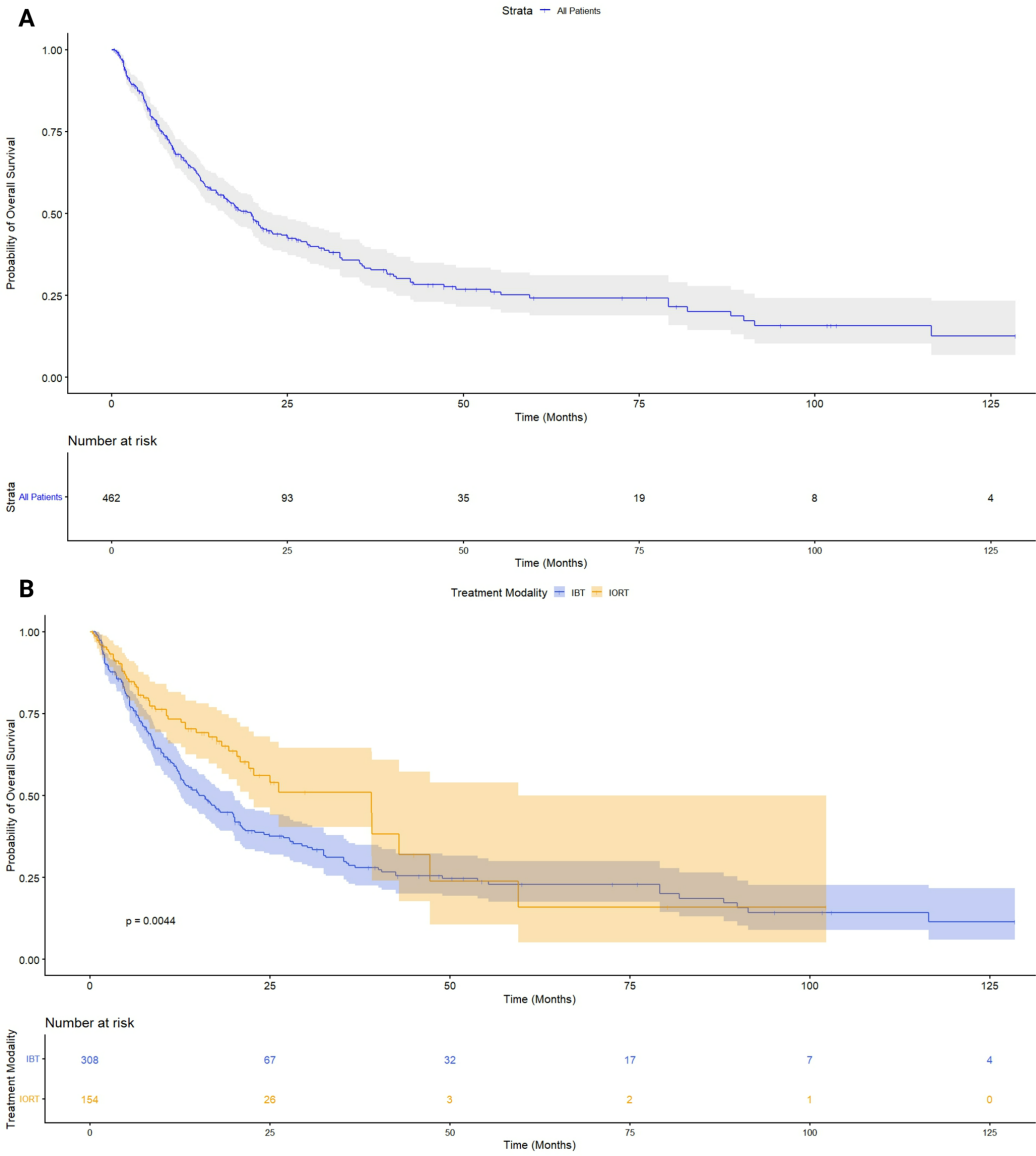
**A** KM survival curve for OS in the entire patient cohort (*n* = 462). The blue line represents the survival probability over time, with shaded areas indicating the 95% confidence interval. The risk table below the curve shows the number of patients at risk at different time points. Figure 2B. KM survival estimates for OS comparing IORT and IBT. The survival probability is plotted over time, with shaded areas representing the 95% confidence intervals. The number at risk at different time points is displayed below the x-axis. Survival curves were reconstructed from published KM plots using IPD extraction. Log-rank test: *p* = 0.0044

KM survival curves were reconstructed using IPD extracted from published survival plots [16, 22, 23, 26, 28, 29, 33, 38]. A total of 462 patients were included. Median OS for the entire cohort was 19.8 months (95% CI: 16.0–22.7). Stratified Kaplan-Meier curves (Fig. 2B) showed a median OS of 39.1 months (95% CI: 22.0–59.5) with IORT, whereas median OS in patients who underwent IBT was 15.9 months (95% CI: 12.6–19.9; log-rank *p* = 0.004).

In univariable Cox analysis, IORT was associated with a significantly lower hazard of death (HR = 0.64, 95% CI: 0.47–0.87, *p* = 0.004).

### Distant brain control

As a secondary outcome, DBC was analyzed across 14 studies (*n* = 625). The pooled one-year DBC rate was 53% (95% CI: 49–57%; I² = 51.6%, *p* = 0.01; supplementary Fig. 1A).

Subgroup analysis showed higher DBC with IORT (57%, 95% CI: 51–62%; I² = 52.1%, *p* = 0.05; 5 studies, *n* = 360) compared to IBT (48%, 95% CI: 42–54%; I² = 33.4%, *p* = 0.18; 9 studies, *n* = 336) (supplementary Fig. 1B–C).

### Leptomeningeal disease and radiation necrosis

The incidence of LMD was analyzed based on 9 studies, encompassing a total of 424 patients. Chen et al. reported a 13.9% LMD rate across mixed tumor histologies [18]. Cummins et al. reported a 39.5% LMD rate, but without specification for the brachytherapy subgroup [19]. The pooled LMD rate across the full cohort was 9% (95% CI: 7–12%; I² = 27%, *p* = 0.19; supplementary Fig. 2A). In subgroup analysis, IORT showed a lower LMD rate (6%, 95% CI: 3–8%; I² = 0.0%, *p* = 0.60; 7 studies, *n* = 360) compared to IBT (9%, 95% CI: 2–16%; I² = 0.0%, *p* = 0.03; 2 studies, *n* = 64) (supplementary Fig. 2B–C).

Radiation necrosis was reported in 20 studies (*n* = 797), with a pooled rate of 5% (95% CI: 4–7%; I² = 53.4%, *p* = 0.01; supplementary Fig. 3A).

IORT showed a lower RN rate (4%, 95% CI: 2–7%; I² = 28.9%, *p* = 0.20; 5 studies, *n* = 360) than IBT (7%, 95% CI: 10–17%; I² = 60.5%, *p* = 0.01; 13 studies, *n* = 437) (supplementary Fig. 3B–C). RN rates were 5% in newly diagnosed cases and 8% in recurrent/mixed cases (see supplementary Fig. 8).

### Local control rate at one year

**Fig. 3:**
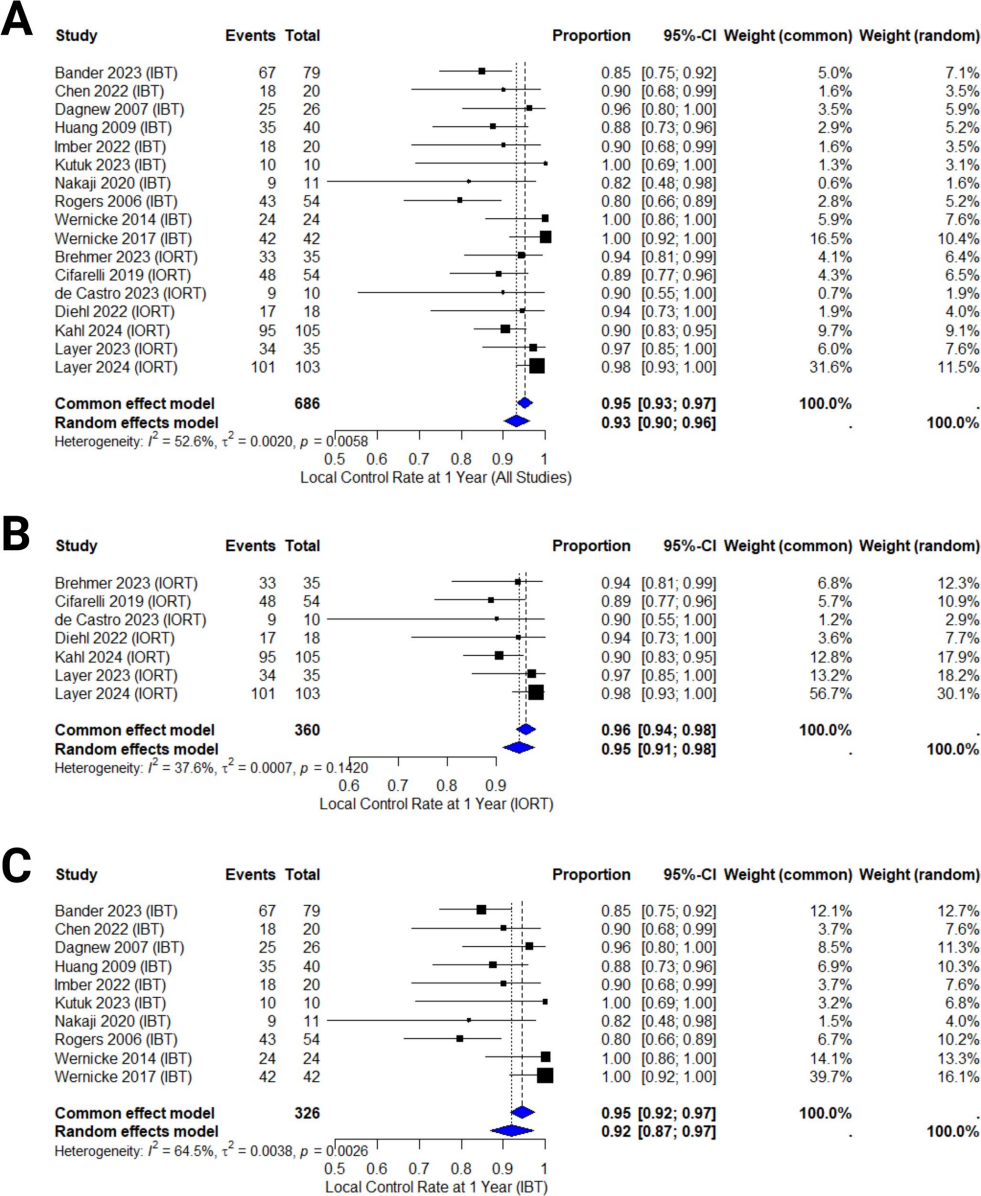
Forest plot displaying the LCR at 1 year for all included studies, based on both common and random effects models. The 95% CI are shown for each study, along with the proportion of local control events. The weight of each study in both fixed and random effects models is also reported. The diamond at the bottom of the figure represents the pooled local control rate estimate for the entire cohort. **3 A** - All studies combined, *p* = 0.01. **3B** - IORT studies, *p* = 0.14. **3 C** - IBT studies, *p* = 0.01

The one-year LCR was analyzed based on data from 17 studies, encompassing a total of 686 patients. The pooled LCR estimate for the entire cohort was 95% (95% CI: 93–97%, see Fig. 3). The heterogeneity among studies was moderate to substantial (I² = 52.6, *p* = 0.01).

A total of 7 studies, including 360 patients, reported LCR outcomes for IORT. The pooled one-year local control rate in the IORT-group was 96% (95% CI: 94–98%) (see Fig. 3B). The heterogeneity within this subgroup was low (I² = 37.6%, *p* = 0.14).

For IBT, 10 studies provided data, comprising 326 patients. The pooled one-year LCR for this subgroup was 95% (95% CI: 92–97%, see Fig. 3C). The heterogeneity in this subgroup was substantial (I² = 64.5%, *p* = 0.01).

Subgroup analysis by tumor histology showed a pooled 1-year LCR of 93% in studies with ≥ 50% lung primaries (*n* = 487; supplementary Fig. 4), and 89% in those with < 50% (*n* = 199; supplementary Fig. 5). IORT studies showed 95% LCR, IBT 89%. Leave-one-out analysis confirmed the robustness of results (supplementary Fig. 6). As far as disease stage is concerned, LCR was 95% for newly diagnosed and 90% for recurrent/mixed metastases (supplementary Fig. 7). Regarding isotope, Cs-131 achieved 94%, I-125 reached 92% LCR (supplementary Fig. 9).

### Reconstructed pooled survival curves and one-stage meta-analysis of local tumor control following intracavitary radiotherapy

**Fig. 4:**
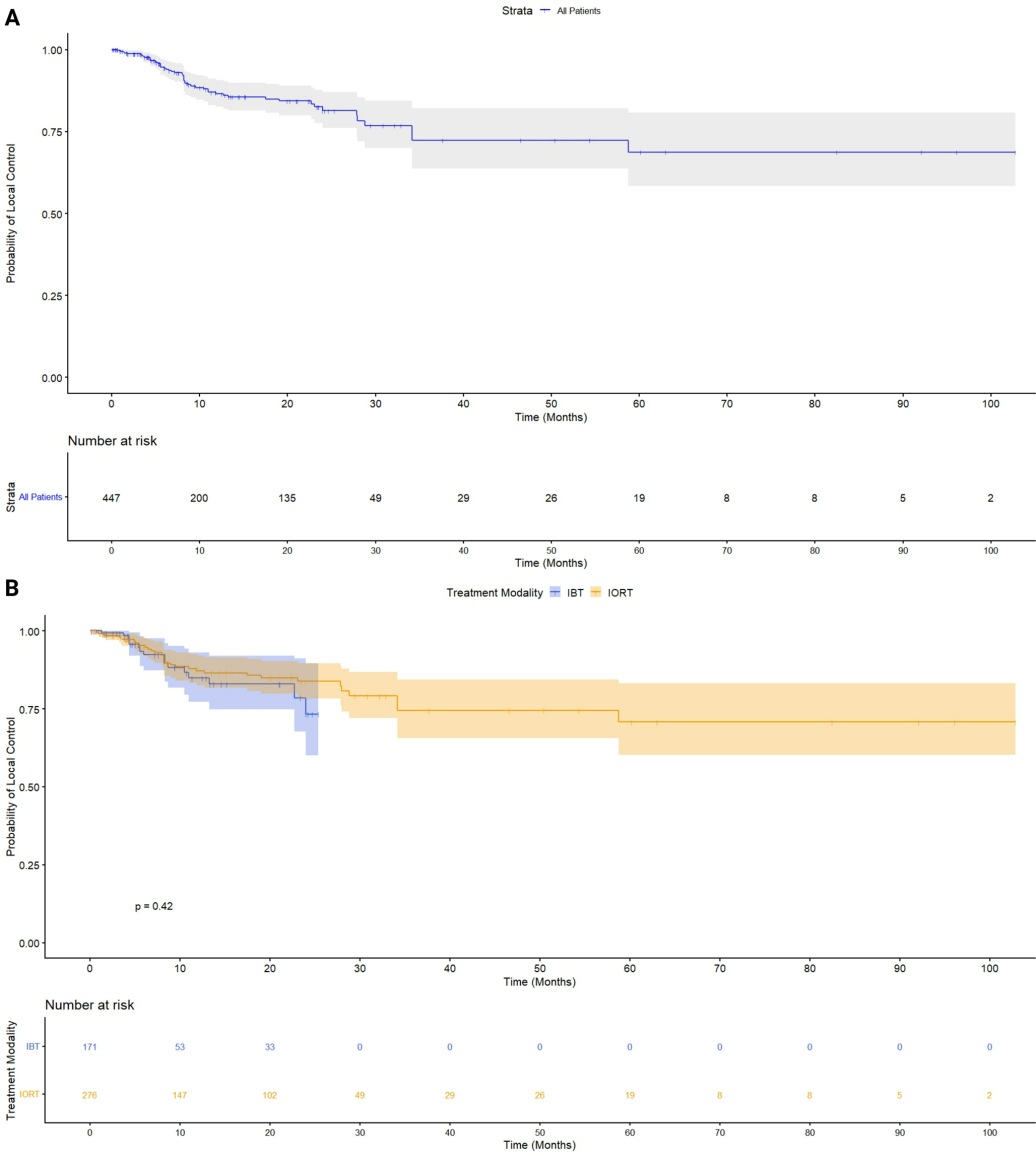
A KM curve for local tumor control in the entire patient cohort (*n* = 447). The blue line represents the probability of local control over time, with shaded areas indicating the 95% confidence interval. The risk table below the curve shows the number of patients at risk at different time points. Figure 4B. KM estimates of local control comparing IORT and IBT. The probability of local control is plotted over time, with shaded areas representing the 95% confidence intervals. The number at risk at different time points is displayed below the x-axis. Log-rank test: *p* = 0.42

To longitudinally evaluate local tumor control following intracavitary radiotherapy, an IPD meta-analysis was conducted using data reconstructed from published KM plots [16, 23, 25, 26, 33, 36]. A total of 447 patients were included (171 IORT [38.3%], 276 IBT [61.7%]; median follow-up: 8.6 months, IQR 4.0–21.0). The pooled KM curve (Fig. 4A) illustrates local tumor control for the full cohort. Subgroup analysis showed no significant difference between IORT and IBT (log-rank *p* = 0.42; Fig. 4B). One-year local control rates were comparable, with only minor divergence after two years and overlapping CIs beyond 36 months.

### Bias and quality evaluation

All 23 studies clearly defined their research question and population; however, only two reported sample size calculations, raising concerns about statistical power. Blinding was rare, with only five studies implementing it. Half addressed confounding factors [12]. Detailed NIH quality assessments are provided in supplementary Tables 2 and 3.

## Discussion

This analysis of 858 patients from 23 studies, including reconstructed individual patient data, presents the most comprehensive evidence on intra-cavitary radiotherapy (IORT & IBT) for resected brain metastases, showing 1-year local control rates of 96.0% for IORT and 95.0% for IBT. Compared to reported SRS control rates of 60–80% in randomized trials, these results are promising and support the need for a randomized trial comparing IORT and SRS with 1-year local control as the primary endpoint [5, 7, 39].

Beyond local control, OS was longer with IORT (39.1 months) than with IBT (15.9 months). However, OS depends on multiple factors (e.g., systemic disease, molecular status), and may be confounded by the heterogenous timepoints of the included studies with strong improvements of adjuvant medical therapy.

### Radiation modalities for resected brain metastases

Postoperative radiotherapy prevents recurrence after brain metastasis resection. IORT delivers 30 Gy using 50 kV X-rays and spherical applicators [33, 35, 36, 38], with reduced doses (16–20 Gy) near critical structures [37]. Monte Carlo planning aids delivery [36, 38]. The procedure adds 7–49 min [38], with MRI follow-up at 24–48 h [34, 36]. IBT implants Cs-131 or I-125 seeds, delivering 60–80 Gy over weeks [18, 24, 25, 29, 40]. Cs-131 is spaced 0.5–1 cm [16, 22, 23]; I-125 uses mesh or adhesives [20, 21, 30]. Prolonged exposure may benefit microscopic disease [24, 25, 28, 29]. Cs-131’s short half-life (9.7 d) lowers long-term toxicity vs. I-125 [41]. RN occurred in up to 26% with I-125, none with Cs-131 [21]. IBT risks include infection, hemorrhage, and seed migration [42–44].

### Local control rate

Preventing local recurrence is essential in managing brain metastases. Recurrence in the resection cavity often leads to neurological symptoms, steroid dependence, and impaired quality of life [45–47]. While WBRT and SRS have traditionally reduced recurrence, concerns about neurotoxicity have prompted interest in IORT and IBT as alternatives [48].

Unlike SRS and WBRT, IORT and IBT deliver radiation intraoperatively, preventing the treatment delays associated with postoperative SRS and reducing early tumor repopulation [49]. They also avoid underdosing due to postoperative cavity shrinkage, which can compromise SRS accuracy [50].

IORT and IBT show high local control rates. Wernicke et al. reported 100% LCR at 12 months with Cs-131 brachytherapy [29], while Xia et al. observed no recurrences in Cs-131-treated patients [31]. Nakaji et al. reported 83% LCR at 12 months using GammaTile [25].

SRS efficacy decreases with larger tumors. Traylor et al. found LCR dropped to 85.1% at 12 months in large lesions [51]. IORT and IBT seem more durable in this context; the INTRAMET trial showed IORT achieving 94.3% LCR at one year in large cavities [32].

Comparisons with WBRT are also important. Kocher et al. found WBRT reduced recurrence from 59 to 27% post-surgery [6]. A meta-analysis by Khan et al. showed better control with combined WBRT + SRS, but at the cost of increased toxicity [52].

Radiobiologically, IORT delivers an intense dose causing rapid DNA damage and possibly stimulating immune responses [50, 53]. IBT applies continuous low-dose radiation, exploiting limited tumor cell repair capacity. Wu et al. showed superior control with salvage IBT over repeat external beam radiation (7.39 vs. 5.51 months) [30], highlighting its role in recurrent settings.

### LMD and radiation necrosis

RN is a known late complication of intracranial radiotherapy, especially with SRS and brachytherapy [54–56]. In this meta-analysis, RN rates varied widely: up to 23% with permanent I-125 implants, while IORT-based studies reported < 5%, mostly asymptomatic or conservatively manageable [21, 36–38]. Imber and Wu showed lower RN rates in IORT and Cs-131 groups compared to matched SRS cohorts [22, 30]. In contrast, older brachytherapy reports described higher RN risk, particularly in recurrent or pre-irradiated cases [26, 27]. A meta-analysis by Choi et al. found 22.4% symptomatic RN in brain metastasis patients, underscoring the toxicity gap between modalities [57].

LMD was less frequent but serious, with reported rates between 3 and 13% [21, 36, 38].

Resection may contribute to LMD, though risk factors remain unclear. RN and LMD are key concerns in brain metastasis care, with IORT potentially offering a safer toxicity profile, especially initially.

### Limitations

This meta-analysis, using both binary and reconstructed longitudinal data, has limitations. Most studies were retrospective, posing risks of selection bias, confounding, and data inconsistency. Significant heterogeneity in patient characteristics, radiotherapy methods, isotopes (I-125 vs. Cs-131), and dosimetry limited comparability. Differences in patient selection, treatment era, and prior therapies may have confounded survival and toxicity outcomes. IORT protocols varied in applicator size, dose, and imaging. Although IPD from Kaplan-Meier curves improved precision, some deviation from original data remains possible. Furthermore, it cannot be excluded that the multicentric retrospective study by Layer et al. [38] may also encompass data from the prospective study [37]. Complication reporting (e.g., RN, LMD) was inconsistent. Most studies originated from high-income countries, introducing potential geographical bias. Missing granular data limited multivariable analysis. Histological stratification was restricted; NSCLC could not be reliably separated from other lung subtypes.

Small tumor volumes (< 4 cm³) predominated, limiting generalizability and preventing volume-based sensitivity analysis. Standardized prospective randomized trials comparing IORT and SRS are needed to strengthen the evidence base.

## Conclusion

Intra-cavitary radiotherapy by IORT or IBT subsequent to surgical resection of BMs provides high local tumor control. Prospective randomized controlled trials are needed to compare IORT with conventional adjuvant stereotactic radiotherapy after surgery for newly diagnosed brain metastases.

## Supplementary Information

Below is the link to the electronic supplementary material.

Supplementary Material 1

Supplementary Material 2

## Data Availability

No datasets were generated or analysed during the current study.
