# Supplementary material for: Intra-cavitary radiotherapy for surgically resected brain metastases: a comprehensive analysis including an individual patient data meta-analysis of intraoperative radiotherapy (IORT) and brachytherapy (IBT)
Source: J Neurooncol. 2025 Sep 17;175(3):907–19. doi: 10.1007/s11060-025-05227-2 (PMC12511155; doi:10.1007/s11060-025-05227-2)
Supplement: Supplementary file 1 — Supplementary Material 1 [file 11060_2025_5227_MOESM1_ESM.docx]

**Supplementary material 1.** PRISMA 2020 Checklist for the Systematic Review and Meta-Analysis on Intra-Cavitary Radiotherapy (IORT vs. IBT) After Brain Metastasis Resection

| Section | Item | Description | Page Number |
| --- | --- | --- | --- |
| Title | Title | A Comprehensive Analysis Including an Individual Patient Data Meta-Analysis of Intraoperative Radiotherapy (IORT) and Brachytherapy (IBT) | 1 |
| Abstract | Structured Summary | A structured abstract summarizing Background, Objectives, Data Sources, Eligibility Criteria, Data Extraction Methods, Synthesis, Results, Limitations, and Conclusions is provided. | 3 |
| Introduction | Rationale | \| The rationale is described in the Background section, highlighting the need for comparative outcome data between IORT and IBT as novel alternatives to SRS or WBRT. \| \| --- \| | 4 |
| Introduction | Objectives | The specific objectives of the study are to quantitatively compare IORT and IBT in terms of local control, overall survival, distant brain failure, and complication rates, using both aggregate and reconstructed individual patient data from 24 studies. | 4-5 |
| Methods | Protocol and registration | The review methodology was predefined and follows PRISMA guidelines. The protocol is registered with PROSPERO (ID: CRD420251026983). | 4 |
| Methods | Eligibility Criteria | Inclusion criteria cover patients with resected brain metastases receiving IORT or IBT, studies with survival and local control data, and published in peer-reviewed journals. | 5 |
| Methods | Information Sources | Databases searched included PubMed, Google Scholar, and Cochrane Library for studies published from 1999 to January 2025, with no language restrictions. | 4-5 |
| Methods | Search Strategy | The search strategy included Boolean terms and keywords related to 'brain metastases', 'intraoperative radiotherapy', and 'brachytherapy'. Detailed syntax is included in the supplementary material. | 5 |
| Methods | Selection Process | Two reviewers independently screened titles and abstracts. Discrepancies were resolved through consensus. | 5 |
| Methods | Data Collection Process | Data extraction was performed using standardized templates. Kaplan-Meier curves were digitized using DigitizeIt and reconstructed using IPDfromKM. | 6 |
| Methods | Data Items | Data were extracted using a standardized form and included study details, patient demographics, tumor characteristics, treatment modality (IORT vs. IBT), and follow-up. Main endpoints were LCR, OS, DBC, LMD, and RN, defined consistently across studies or derived when missing. | 6 |
| Methods | Study Risk of Bias Assessment | Risk of bias evaluation using the NIH Quality Assessment Tool for Observational Cohort and Cross-Sectional Studies (NIH-QAT) was performed. | Supplementary Pages 7–8 |
| Methods | Effect Measures | Effect measures included Kaplan-Meier survival rates, 1-year local control, hazard ratios, and pooled incidence rates. | 6 |
| Methods | Synthesis of Results | Pooled analyses were conducted using random-effects meta-analysis. Heterogeneity was assessed via I² statistics. | 6 |
| Methods | Risk of Bias Across Studies | Summary of risk of bias across studies based on NIH-QAT findings is given. | 5-6 |
| Results | Study Selection | The selection process is outlined using a PRISMA flow diagram detailing the number of records identified, screened, and included. | 7 |
| Results | Study Characteristics | Study-level characteristics such as design, sample size, treatment modality, and follow-up were summarized in supplementary Table 1. | 8 |
| Results | Results of Individual Studies | Key characteristics of the included studies (e.g., author, year, country, design, patient number, demographics, tumor location, histopathology, and follow-up duration) are summarized in Supplementary Table 1. The table includes both IORT and IBT cohorts and is referenced in the results section. | Supplementary Pages 1–6 |
| Results | Synthesis of Results | Meta-analyses were conducted separately for IORT and IBT using reconstructed individual patient data and reported outcomes. Pooled estimates for 1-year local control, overall survival, radiation necrosis, distant brain failure, and leptomeningeal disease were calculated using random-effects models with inverse variance weighting. Heterogeneity was assessed with I² and τ² statistics. | 8-13 |
| Results | Risk of Bias in Studies | A summary of the risk of bias across included studies is provided and discussed. | 13 |
| Discussion | Summary of Evidence | The discussion summarizes pooled outcome patterns for IORT and IBT, highlighting comparative trends in local control, survival, and complication rates, and interprets findings in light of clinical applicability and prior studies. | 14-16 |
| Discussion | Limitations | The review acknowledges limitations including retrospective design, heterogeneity in techniques and dosimetry, reliance on reconstructed data, inconsistent reporting of complications, and lack of multivariable adjustments. | 16 |
| Discussion | Conclusions | Conclusions emphasize the feasibility and safety of intra-cavitary techniques and support further randomized trials. | 16 |
| Funding | Funding | The manuscript discloses no external funding and provides a conflict of interest statement. | 16 |
| Acknowledgments | Contributions | Acknowledge any additional contributions by authors and creation tools (e.g., BioRender for visual abstracts). | 17 |
