# Supplementary material for: Intra-cavitary radiotherapy for surgically resected brain metastases: a comprehensive analysis including an individual patient data meta-analysis of intraoperative radiotherapy (IORT) and brachytherapy (IBT)
Source: J Neurooncol. 2025 Sep 17;175(3):907–19. doi: 10.1007/s11060-025-05227-2 (PMC12511155; doi:10.1007/s11060-025-05227-2)
Supplement: Supplementary file 2 — Supplementary Material 2 [file 11060_2025_5227_MOESM2_ESM.docx]

| **Supplementary Table 1.** Summary of Systematic Review of Studies on Intracavitary Radiotherapy for Brain Metastases Regarding Treatment Approaches and Study Characteristics. | | | | | | | | | | | | | |
| --- | --- | --- | --- | --- | --- | --- | --- | --- | --- | --- | --- | --- | --- |
| **Author** | **Year** | **Country** | **Study design** | **Type of intracavitary radiotherapy** | **No. of patients** | **Median KPS** | **Median / Mean Age** | **Median Size / Volume** | **Localization** | **Primary or recurrent treatment** | **No. of metastases** | **Primary histopathology** | **Median / Mean Follow-Up** |
| Bander [16] | 2023 | USA | Retrospective | IBT | 79 |  | 62.6 * | 3.00 cm* | - | 49 New  30 Recurrent | 79 | NSCLC (n = 45) Melanoma (n = 5) Breast (n = 5)  Renal (n = 5)  GI (n = 15)  Cervical (n = 1)  Endometrial (n = 1)  Other (n = 2) | 11.8 * |
| Bogart [17] | 1999 | USA | Retrospective | IBT | 15 | 70 |  | 2.50 cm | Frontal (5), Parietal (5), Occipital (4), Temporal (1) | 15 New | 15 | NSCLC (n = 15) | 14.0 |
| Brehmer [33] | 2023 | Germany | Prospective | IORT | 35 |  | 64.0 |  | - | 35 New |  | Lung (68.6 %) | 25.70 |
| Chen [18] | 2022 | USA | Retrospective | IBT | 20 | 80 * | 58.5 * | 3.00 cm* | - | 20 Recurrent | 22 | NSCLC (n = 5)  Breast (n = 5)  GI (n = 3)  Other (n = 9) | 17 * |
| Cifarelli [34] | 2019 | USA Germany | Retrospective | IORT | 54 |  | 64.0 | - | Frontal (22), Parietal (15), Occipital (6), Temporal (5)  Posterior fossa (6) | 54 New | 54 | NSCLC (n = 23) Melanoma (n = 8) Breast (n = 8) Renal (n = 4)  GI (n = 4)  Gynecologic (n = 2)  Other (n = 5) | 7.2 |
| Cummins [19] | 2022 | USA | Retrospective | IBT | 16 | 80 ** | 58.1 ** | - | - | 16 Recurrent |  |  | 11.1 ** |
| Dagnew [20] | 2007 | USA | Retrospective | IBT | 26 | 90 | 55.0 | 3.00 cm | - | 26 New | 26 | Lung (n = 12) Melanoma (n = 4) Colon (n = 3)  Breast (n = 2)  Renal (n = 1)  Cervix (n = 1)  Prostate (n = 1)  Ovarian (n = 1)  Unknown (n = 1) | 12.0 |
| de Castro [35] | 2023 | Brazil USA Germany | Retrospective | IORT | 10 |  | 58.0 | 3.10 cm | - | 10 New | 10 | NSCLC (n = 4) Melanoma (n = 2) Breast (n = 2)  Colon (n = 1)  Endometrium (n =1) | 11.2 |
| Diehl [36] | 2022 | Germany | Retrospective | IORT | 18 | 85 | 56.0 |  | Frontal (7), Parietal (7), Occipital (2), Temporal (2) | 18 New | 18 | NSCLC (n = 6)  Melanoma (n = 4)  RCC (n = 2)  TNBC (n = 1) BC (n = 1)  Osteosarcoma (n =1)  Pancreatic (n = 1) Urothelial (n = 1)  Rectal (n = 1) | 10.80 |
| Huang [21] | 2008 | USA The Netherlands | Retrospective | IBT | 40 | 80 | 59.0 | 3.20 cm | Frontal (11), Parietal (7), Occipital (4), Temporal (11)  Posterior fossa (5) | 19 New 21 Recurrent |  | Lung (n = 18) Melanoma (n = 16) Breast (n = 3) Other (n = 3) | 73.2 |
| Imber [22] | 2022 | USA | Prospective | IBT | 20 |  | 59.2 | 3.00 cm | Frontal (7), Parietal (7), Occipital (2), Temporal (4)  Posterior fossa (5) | 20 Recurrent | 25 | Lung (n = 5) Breast (n = 4) Renal (n = 3)  Other (n = 8) | 19.2 |
| Julie [23] | 2021 | USA | Retrospective | IBT | 30 | 80 | 62.0 | 2.70 cm | Frontal (8), Parietal (10), Occipital (2), Temporal (2)  Posterior fossa (7) | 30 New | 30 | NSCLC (n = 18) Breast (n = 3)  GI (n = 5)  Other (n = 4) | 17.5 |
| Kahl [37] | 2024 | Germany | Retrospective | IORT | 105 |  | 65.0 | 3.10 cm | Frontal (33), Parietal (25), Occipital (26), Temporal (18), Posterior fossa (15) | 105 New | 117 | NSCLC (n = 50) Melanoma (n = 16) Breast (n =13) Renal (n = 8)  SCLC (n = 4)  Other (n = 17) | 14.0 |
| Kutuk [24] | 2023 | USA | Retrospective | IBT | 10 | 90 | 56.0 | 2.70 cm | Frontal (3), Parietal (5), Occipital (1), Temporal (1) | 10 Recurrent | 12 | Lung (n = 5) Melanoma (n = 1) Breast (n = 4) Renal (n = 1)  Colorectal (n = 1) | 14.5 |
| Layer [39] | 2024 | Germany  Brazil | Retrospective | IORT | 103 | 80 | 63.0 | 22.90 cm³ | Frontal (36), Parietal (28), Occipital (18), Temporal (13), Posterior fossa (8) | 103 New | 103 | NSCLC (n = 53) Melanoma (n = 25) Breast (n = 4) RCC (n = 13)  SCLC (n = 3)  Other (n = 5) | 13.2 |
| Layer [38] | 2023 | Germany | Prospective | IORT | 35 | 80 | 63.0 |  | - | 35 New |  | Lung (n = 21) Melanoma (n = 4) Breast (n = 2) Renal (n = 4)  Other (n = 4) | 10.4 |
| Nakaji [25] | 2020 | USA | Prospective | IBT | 11 | 70 | 60.0 | 3.20 cm | Frontal (4), Parietal (7), Temporal (3), Posterior fossa (2) | 4 New 12 Recurrent | 16 | NSCLC (n = 5) Breast (n = 7)  Sarcoma (n = 3)  SCLC (n = 1) | 9.3 |
| Raleigh [27] | 2016 | USA | Retrospective | IBT | 95 | 80 | 59.4 | 13.50 cm³ | Frontal (32), Parietal (17), Occipital (17), Temporal (26), Posterior fossa (13) | 49 New 56 Recurrent | 105 | Lung (n = 36) Melanoma (n = 26) Breast (n =22)  Other (n = 11) | 14.4 |
| Rogers [28] | 2006 | USA | Prospective | IBT | 54 | 90 | 60.0 | 14 cm³ | Frontal (11), Parietal (7), Occipital (4), Temporal (11)  Posterior fossa (5) | 12 New 42 Recurrent |  | Lung (n = 29) Melanoma (n = 7) Renal (n = 3)  GI (n = 7)  Other (n = 8) | - |
| Wernicke [30] | 2014 | USA | Prospective | IBT | 24 |  | 65.0 | 10.31 cm³ | Frontal (15), Parietal (12), Occipital (7), Temporal (6) | 21 New  3 (prior RT) |  | Lung (n = 16) Melanoma (n = 2) Breast (n = 2) Renal (n = 2)  Other (n = 2) | 9.5 |
| Wernicke [29] | 2017 | USA | Prospective | IBT | 42 |  | 65.0 | 3.00 cm | Frontal (14), Parietal (14), Occipital (3), Temporal (4)  Posterior fossa (11) | 32 New  10 (prior RT) | 46 | Lung (n = 26) GI (n = 7)  Breast (n = 3)  Other (n = 6) | 11.9 |
| Wu [31] | 2022 | USA | Retrospective | IBT | 13 |  | 62.0 |  | Frontal (4), Parietal (4), Occipital (2), Temporal (3)  Posterior fossa (1) | 13 Recurrent |  | Lung (n =8) Melanoma (n = 2) Breast (n = 2) | 7.2 |
| Xia [32] | 2018 | USA | Retrospective | IBT | 11 |  | 53.8 | 3.00 cm | Frontal (4), Parietal (1), Occipital (3), Temporal (1) | 9 Recurrent |  | Lung (n = 2) Melanoma (n = 1) Breast (n = 3)  Other (n = 3) | 10.3 |

*, ** Includes patients not only with brain metastases, but also other entities within the total cohort.

| **Supplementary Table 2. NIH Quality Assessment of Retrospective Studies (Part 1).** This table presents the quality assessment of various retrospective studies based on NIH criteria. Each study was evaluated across multiple methodological parameters. Studies were categorized as either meeting (“Yes”) or not meeting (“No”) each criterion, providing an overview of their methodological strengths and limitations. | | | | | | | | | | | | |
| --- | --- | --- | --- | --- | --- | --- | --- | --- | --- | --- | --- | --- |
| **NIH Criteria** | **Bander** [16] | **Bogart** [17] | **Brehmer** [33] | **Chen** [18] | **Cifarelli** [34] | **Cummins** [19] | **Dagnew** [20] | **de Castro** [35] | **Diehl** [36] | **Huang** [21] | **Imber** [22] | **Julie** [23] |
| Clearly stated research question | Yes | Yes | Yes | Yes | Yes | Yes | Yes | Yes | Yes | Yes | Yes | Yes |
| Defined study population | Yes | Yes | Yes | Yes | Yes | Yes | Yes | Yes | Yes | Yes | Yes | Yes |
| ≥50% participation rate | Yes | Yes | Yes | Yes | Yes | Yes | Yes | Yes | Yes | Yes | Yes | Yes |
| Uniform inclusion/exclusion criteria | Yes | Yes | Yes | Yes | Yes | Yes | Yes | Yes | Yes | Yes | Yes | Yes |
| Justification of sample size | No | No | No | No | No | No | No | Yes | No | No | No | Yes |
| Exposure assessed before outcome | Yes | Yes | Yes | Yes | Yes | Yes | Yes | Yes | Yes | Yes | Yes | Yes |
| Sufficient follow-up period | Yes | Yes | Yes | Yes | Yes | Yes | Yes | Yes | Yes | Yes | Yes | Yes |
| Measurable/consistent exposure assessment | Yes | Yes | Yes | Yes | Yes | Yes | Yes | Yes | Yes | Yes | Yes | Yes |
| Clearly defined outcome measures | Yes | Yes | Yes | Yes | Yes | Yes | Yes | Yes | Yes | Yes | Yes | Yes |
| Blinded outcome assessment | No | No | No | No | No | No | No | No | No | No | Yes | No |
| ≤20% loss to follow-up | Yes | Yes | Yes | Yes | Yes | Yes | Yes | Yes | Yes | Yes | Yes | Yes |
| Confounders measured and adjusted | Yes | Yes | Yes | Yes | Yes | Yes | Yes | Yes | Yes | Yes | Yes | Yes |

| **NIH Criteria** | **Kahl** [37] | **Kutuk** [24] | **Layer**  [39] | **Layer**  [38] | **Nakaji** [25] | **Raleigh** [27] | **Rogers** [28] | **Wernicke**  [30] | **Wernicke**  [29] | **Wu** [31] | **Xia** [32] |
| --- | --- | --- | --- | --- | --- | --- | --- | --- | --- | --- | --- |
| Clearly stated research question | Yes | Yes | Yes | Yes | Yes | Yes | Yes | Yes | Yes | Yes | Yes |
| Defined study population | Yes | Yes | Yes | Yes | Yes | Yes | Yes | Yes | Yes | Yes | Yes |
| ≥50% participation rate | Yes | Yes | Yes | Yes | Yes | Yes | Yes | Yes | Yes | Yes | Yes |
| Uniform inclusion/exclusion criteria | Yes | Yes | Yes | Yes | Yes | Yes | Yes | Yes | Yes | Yes | Yes |
| Justification of sample size | No | No | No | No | No | Yes | No | No | No | No | No |
| Exposure assessed before outcome | Yes | Yes | Yes | Yes | Yes | Yes | Yes | Yes | Yes | Yes | Yes |
| Sufficient follow-up period | Yes | Yes | Yes | Yes | Yes | Yes | Yes | Yes | Yes | No | No |
| Measurable/consistent exposure assessment | Yes | Yes | Yes | Yes | Yes | Yes | Yes | Yes | Yes | Yes | Yes |
| Clearly defined outcome measures | Yes | Yes | Yes | Yes | Yes | Yes | Yes | Yes | Yes | Yes | Yes |
| Blinded outcome assessment | No | No | No | No | No | No | Yes | Yes | Yes | Yes | No |
| ≤20% loss to follow-up | Yes | Yes | Yes | Yes | Yes | Yes | Yes | Yes | Yes | Yes | Yes |
| Confounders measured and adjusted | No | No | No | No | No | No | No | No | No | No | No |


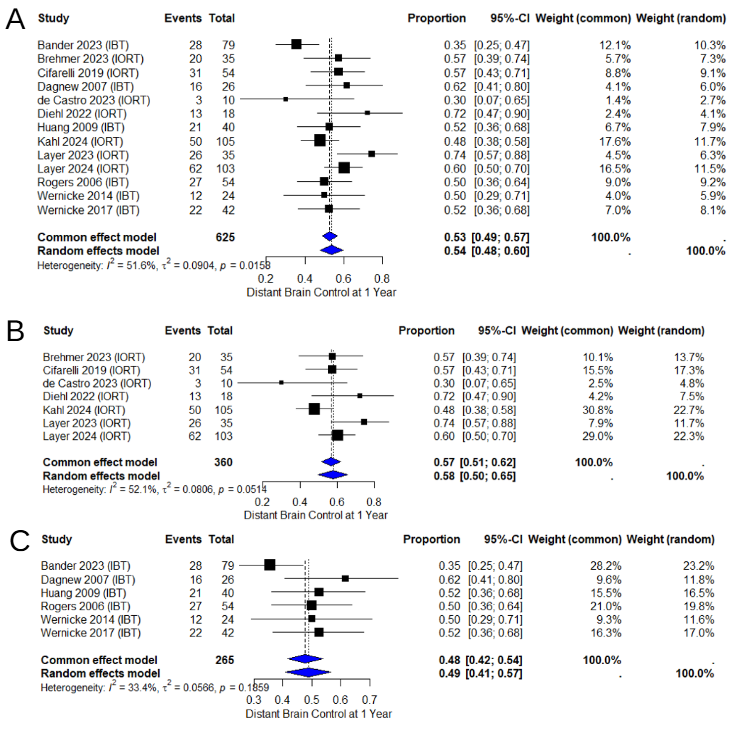


**Supplementary Figure 1.** Forest plot displaying the one-year DBC for all included studies, based on both common and random effects models. The 95% CI are shown for each study, along with the proportion of distant brain control events. The weight of each study in both fixed and random effects models is also reported. The diamond at the bottom of the figure represents the pooled DBC estimate for the entire cohort. **1A** - All studies combined, *p* = 0.01. **1B** - IORT studies, *p* = 0.05. **1C** - IBT studies, *p* = 0.18.


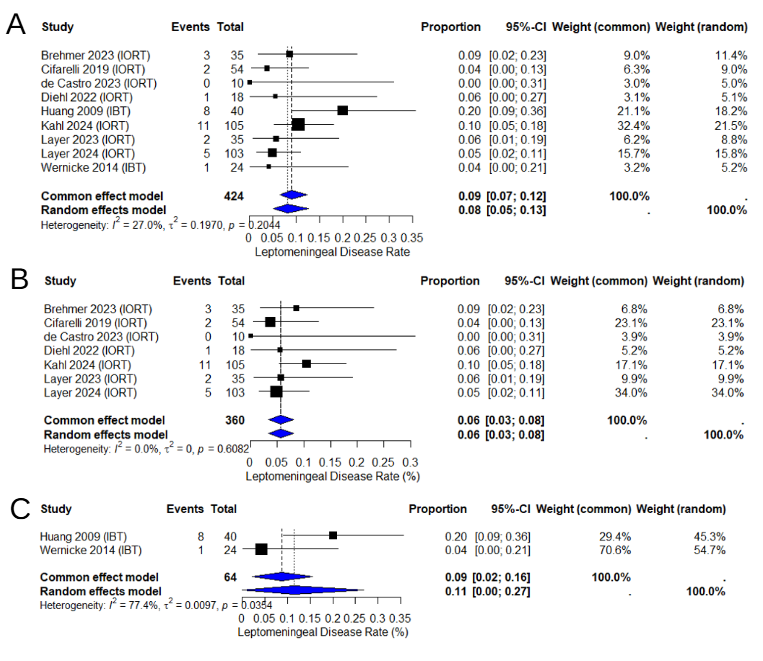


**Supplementary Figure 2.** Forest plot showing the LMD progression for all included studies. The 95% CI for each study are indicated along with the proportion of LMD events. The weight of each study is provided for both the common and random effects models. The diamond at the bottom of the plot represents the pooled estimate of LMD progression for the entire cohort. **2A** - All studies combined, *p* = 0.19. **2B** – IORT studies, *p* = 0.60. **2C** – IBT studies, *p* = 0.03.

**
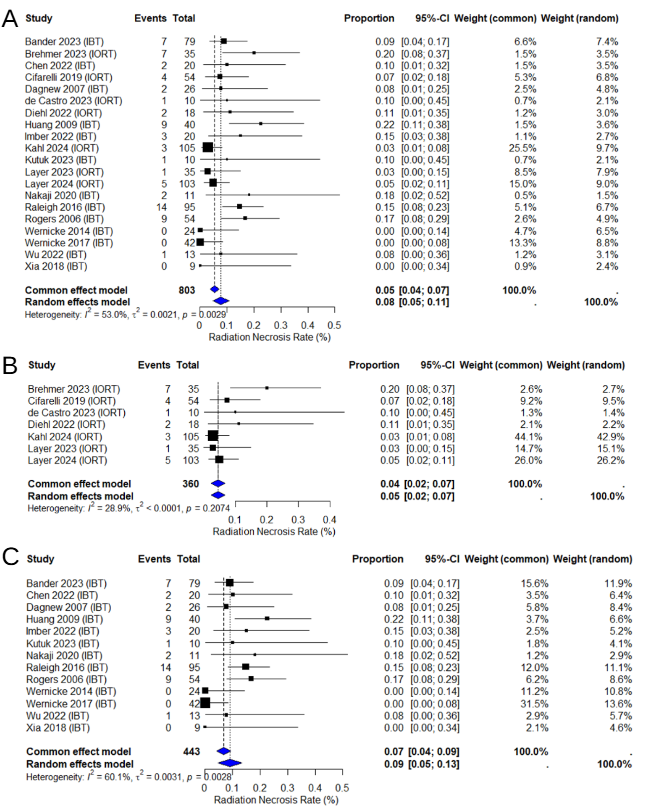
**

**Supplementary Figure 3.** Forest plot displaying the radionecrosis rate for all included studies, based on both common and random effects models. The 95% CI are shown for each study, along with the proportion of radionecrosis events. The weight of each study in both fixed and random effects models is also reported. The diamond at the bottom of the figure represents the pooled radionecrosis rate estimate for the entire cohort. **3A** - All studies combined, *p* = 0.01. **3B** - IORT studies, *p* = 0.20. **3C** - IBT studies, *p* = 0.01.


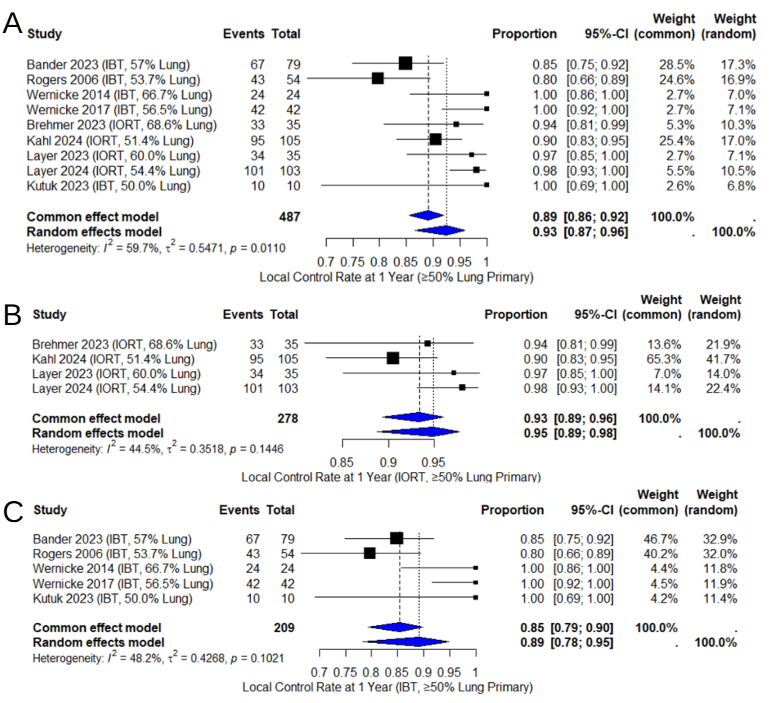


**Supplementary Figure 4.** Forest plot displaying the LCR at 1 year in studies including ≥50% of patients with a lung primary tumor, based on both common and random effects models. The 95% CI are shown for each study, along with the proportion of local control events. The weight of each study in both fixed and random effects models is also reported. The diamond at the bottom of each panel represents the pooled local control rate estimate for the respective subgroup. **4A** – All studies combined, *p* = 0.01. **4B** – IORT studies, *p* = 0.14. **4C** – IBT studies, *p* = 0.01.

*Note: In Kutuk 2023, the lung primary percentage (50.0%) may have been based on metastasis counts rather than patient-level data.*


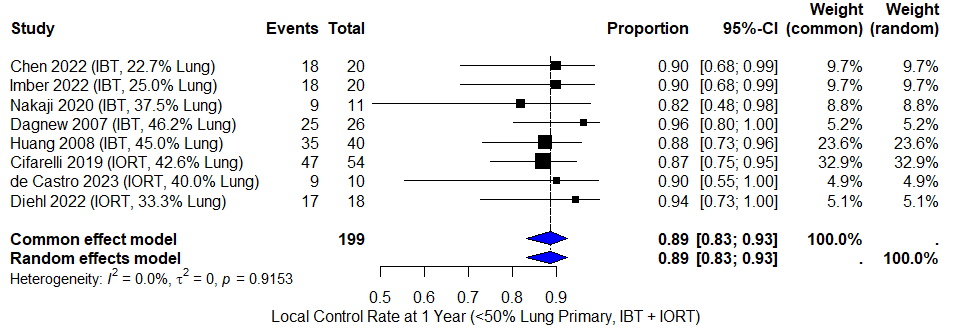


**Supplementary Figure 5.** Forest plot of the 1-year LCR in studies (IBT and IORT) including <50% of patients with a lung primary tumor. Each study is shown with its estimated proportion, 95% CI, and statistical weights under both the fixed-effect and random-effects models. The pooled LCR is indicated by blue diamonds at the bottom. The random-effects model yielded a pooled estimate of 0.89 [95% CI: 0.83–0.93], with no observed heterogeneity (*I²* = 0.0%, τ² = 0, *p* = 0.9153).

*Note: In Nakaji 2020, the lung primary percentage (37.5%) was based on metastasis-level data (16 lesions in 11 patients); patient-level proportions could not be determined.*


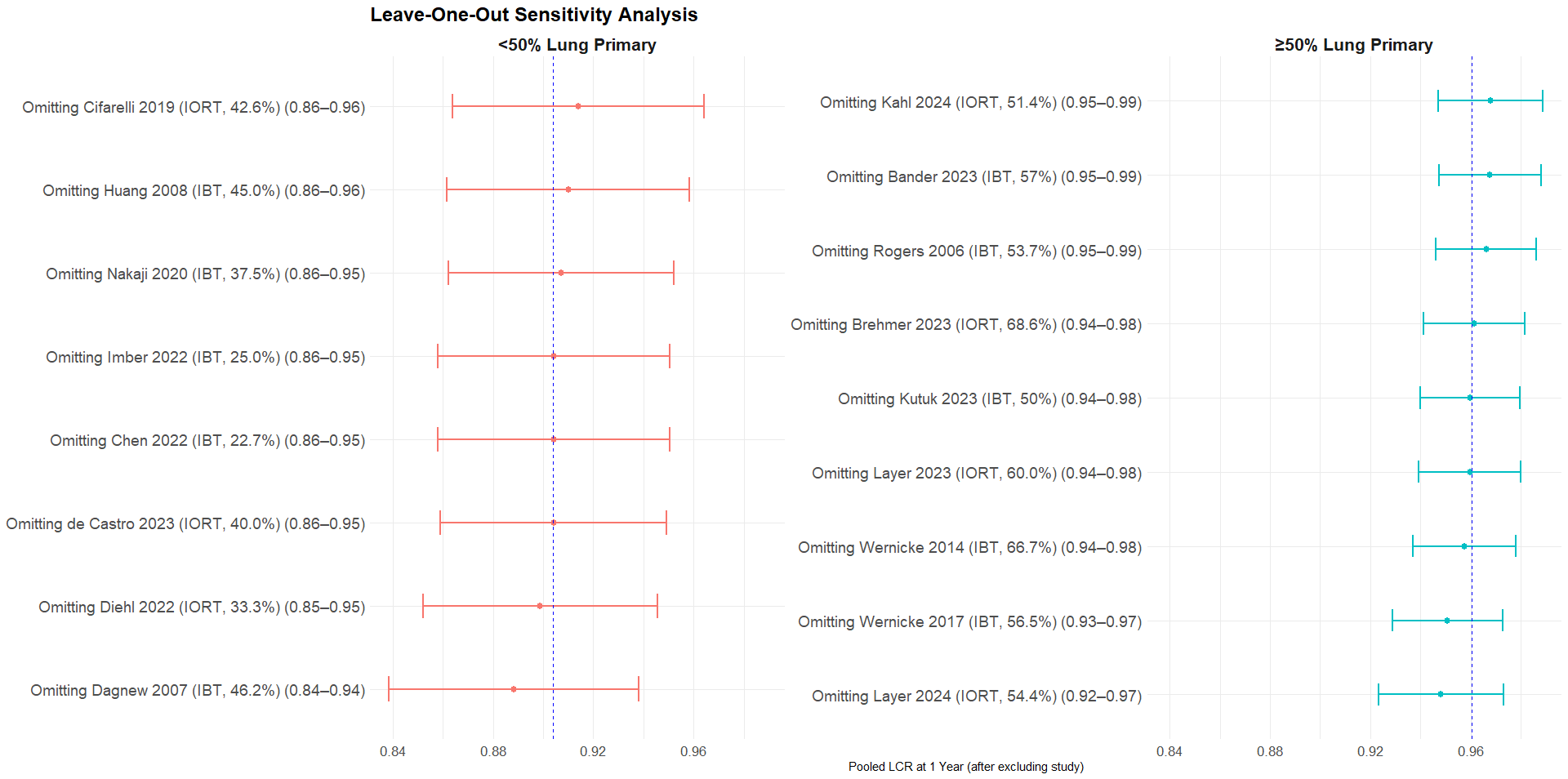


**Supplementary Figure 6.** Leave-one-out sensitivity analysis for the 1-year LCR. Each data point represents the pooled LCR estimate after exclusion of one study. Blue confidence intervals indicate studies including ≥50% of patients with a lung primary tumor, whereas red intervals denote studies with <50% lung primaries. The vertical dashed blue line marks the overall pooled LCR across all studies. The red dashed lines indicate the subgroup-specific pooled estimates for the <50% and ≥50% lung primary groups, respectively.


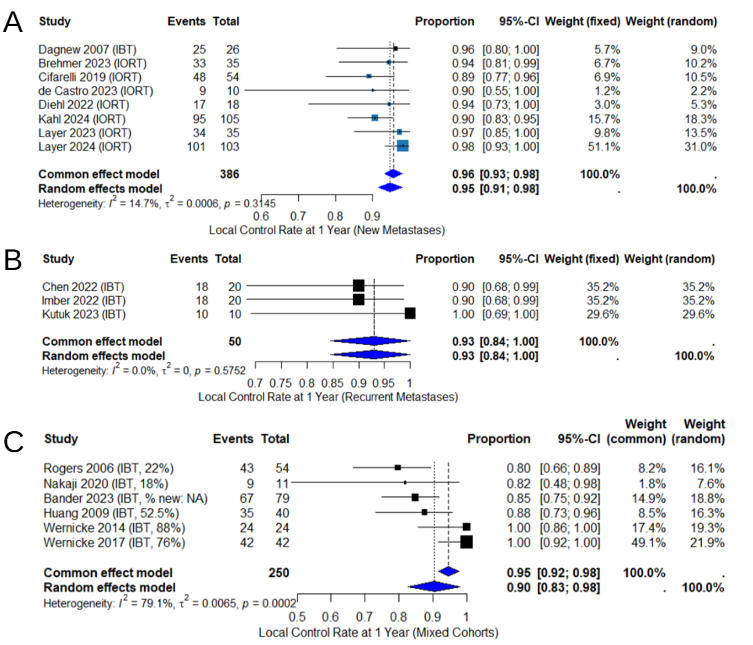


**Supplementary Figure 7.** Forest plot displaying the 1-year LCR for all included studies, calculated using both common-effect and random-effects models. Each study is represented with its estimated proportion of local control events, corresponding 95% CI, and statistical weights under both models. Diamonds at the bottom indicate the pooled effect estimates, with their width corresponding to the 95% CI. **7A** – All studies of new metastases, *p* = 0.3145. **7B** – Studies of recurrent metastases, *p* = 0.5752. **7C** – Mixed-cohort studies including both new and recurrent metastases, *p* = 0.0002.

***Note (7C):*** *In panel C, the percentages in parentheses next to each study reflect the proportion of patients with newly diagnosed brain metastases, where available. In Bander 2023, the exact basis for this percentage was not specified and may not be patient-level data.*


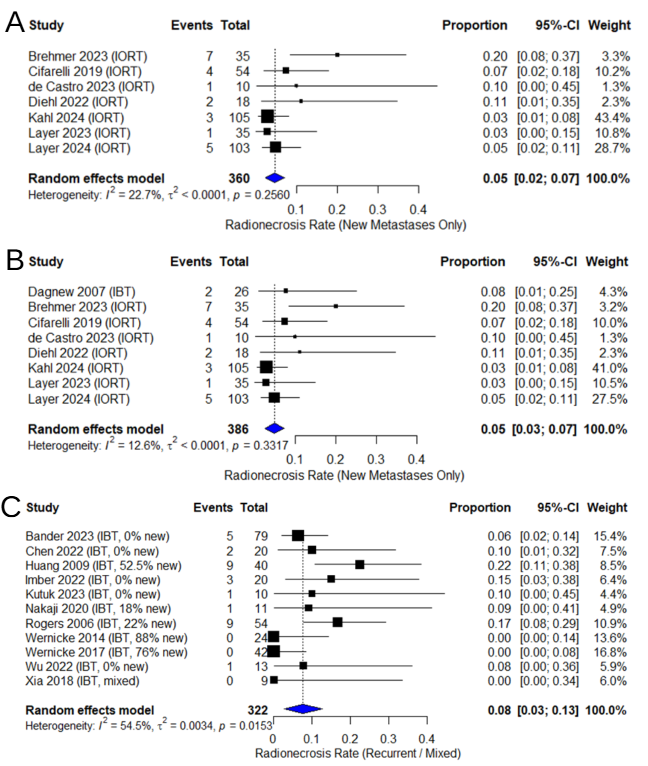


**Supplementary Figure 8.** Forest plot displaying the radionecrosis rate in patients with brain metastases, calculated using random-effects models. Each study is represented by its estimated proportion of radionecrosis events, corresponding 95% CI, and statistical weights. Diamonds at the bottom indicate the pooled effect estimates, with their width corresponding to the 95% CI. 8**A** – Studies of newly diagnosed metastases treated with IORT, *p* = 0.2560. **8B** – Studies of newly diagnosed metastases treated with IORT or IBT, *p* = 0.3317. **8C** – Studies of recurrent or mixed metastases treated with IBT, *p* = 0.0153.

***Note (8C):*** *In panel C, the percentages in parentheses after each study name indicate the proportion of patients with newly diagnosed metastases.*

*
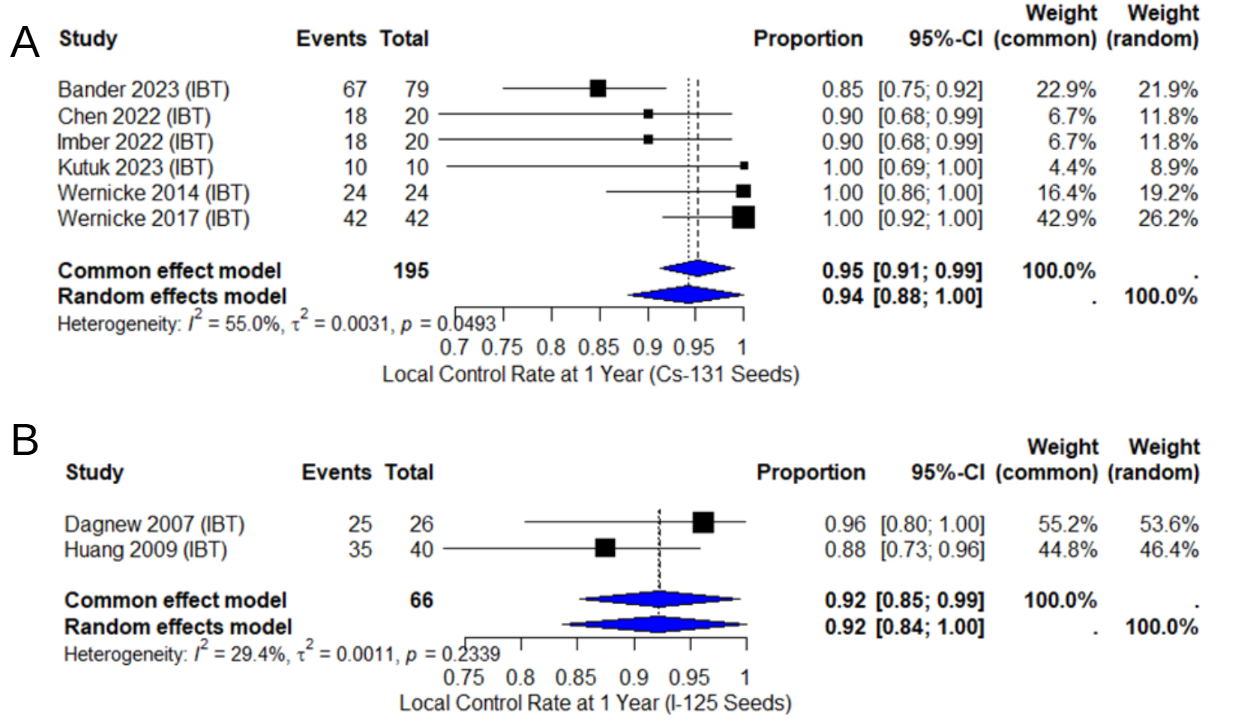
*

**Supplementary Figure 9.** Forest plot displaying the 1-year LCR for patients with brain metastases treated with permanent interstitial brachytherapy, using either Cesium-131 or Iodine-125 seeds. Proportions with corresponding 95% CI are shown for each study, and pooled effect estimates are calculated using both common-effect and random-effects models. Diamonds represent the summary effects, with their width indicating the 95% CI. **9A** – Studies using Cs-131 seeds, *p* = 0.0493. **9B** – Studies using I-125 seeds, *p* = 0.2339.
